# *Nuttalliella namaqua* (Ixodoidea: Nuttalliellidae): First Description of the Male, Immature Stages and Re-Description of the Female

**DOI:** 10.1371/journal.pone.0041651

**Published:** 2012-07-26

**Authors:** Abdalla A. Latif, John F. Putterill, Daniel G. de Klerk, Ronel Pienaar, Ben J. Mans

**Affiliations:** 1 Parasites, Vectors and Vector-borne Diseases Programme, Agricultural Research Council–Onderstepoort Veterinary Institute, Onderstepoort, South Africa; 2 Department of Veterinary Tropical Diseases, Faculty of Veterinary Science, University of Pretoria, Onderstepoort, South Africa; Universidade Federal do Rio de Janeiro, Brazil

## Abstract

*Nuttalliella namaqua* is the only species of the enigmatic third tick family. Females possess features of hard and soft ticks and have been designated as the “missing link” between the main tick families. Its position at the base of the tick tree suggests that some of the features unique to hard and soft ticks were present in the ancestral tick lineage. Larvae, nymphae and males have not been described to date and questions regarding their biological affinities to the main tick families remain unclear. The current study addressed these questions via the description of larvae, nymphae and males and resolved issues pertaining to female morphology. Field collected as well as laboratory-engorged females laid eggs and viable larvae subsequently hatched. The larvae possess morphological structures not present in subsequent stages: namely, a sclerotized scutum, pores on the dorsal surface of legs and a dentate anal plate. The last two characters are not present in ixodids and argasids. *N. namaqua* larvae and nymphae show a similar morphology to females: a unique hypostomal structure i.e., bluntly rounded apically in nymphae and females and ball-like in the larvae. A re-description of some structures in female *N. namaqua* has resolved differences in the original descriptions, namely that *N. namaqua* have 4 palpal segments as found in ixodids and argasids and posthypostomal setae. The male was discovered for the first time and described. Characteristic male features include: a pseudoscutum over most of the dorsum, an outgrowth on the chelicerae forming a unique rod-like structure similar to a spematodactyl in mites and medial extension of palpal segment 2 forming a large ventral crib for segment 4. All life stages possess some features found in hard and soft ticks and its status as the “missing link” between the tick families remains.

## Introduction

Ticks are divided into three families, the Ixodidae (hard ticks), Argasidae (soft ticks) and Nuttalliellidae (monotypic) [1]. Hard and soft ticks differ in their morphology and biology. Ixodids in all life stages have a sclerotized scutum, an apical hypostome, feed for prolonged periods, ingest more than hundred times their own mass in blood and concentrate the blood meal by secreting water via their salivary glands [2]–[3]. Soft ticks have a leathery integument and lack a scutum, their hypostome is located anterior ventrally, feed for short periods and concentrate the blood meal by secreting water via the coxal organs [2]–[3]. *N. namaqua* shares several characteristics with both hard and soft tick families and has been described as the “missing link” between the tick families [4]–[5]. Systematic analysis based on the 18 S ribosomal RNA indicated that ticks are monophyletic and that *N. namaqua* grouped basal to the other families, suggesting a close relationship to the ancestral tick lineage [6]. Its study could therefore shed light on the evolution of the other tick families.

The original description of *N. namaqua* was based on a single engorged female using light microscopy [4]. This study showed that *N. namaqua* had a leathery integument, rudimentary hypostomal teeth and that the fourth segment of the palps was terminal as found in argasids, but possess a pseudoscutum and apical hypostome reminiscent of ixodids [4]. No spiracle could be discerned in the latter study. Forty-five years later, *N. namaqua* was rediscovered in Tanzania and re-described using scanning electron microscopy of a single female specimen, although fourteen of the fifteen available specimens were also examined using light microscopy [7]. It was indicated that characteristics that relate *N. namaqua* to ixodids included the apical position of the capitulum, its pseudoscutum, absence of a ventral paired organ, coxal and supra coxal folds, and the similarity of dorsal and ventral integuments [7]. Characteristics that relate *N. namaqua* to argasids were the integumental structure, unarmed coxae, hypostomal structure and lack of porose areas. Unique characteristics included the organs of unknown function posterior to coxae IV, three segmented palpi, pseudoscutum and ball and socket leg joints, Haller’s organ structure and lack of spiracle plates [7]. The organ of unknown function was subsequently shown to be the spiracle with a unique fenestrated spiracle plate [8]. The spiracle plate possesses argasid features, notably, the lack of an elevated well-defined marginal peritreme as found in ixodids. Several features are similar to ixodids, namely, the macula that is located anteriorly and forms a thickened lip that encloses the crescentic ostium, the ostial lip that contains a luminal extension and the valve-like projection between the subostial space and atrial chamber [8].

The internal morphology of *N. namaqua* also shows features similar to both argasids and ixodids [9]. The stomach lobe numbers and arrangement, the route of the Malpighian tubules through the organs, the transverse position of the ovary, bilobed uterus, vagina diversion into cervical and vestibular parts, absence of a vaginal chamber and seminal receptacle and the number and disposition of main tracheal trunks are shared with the argasids. The unlobed rectal sac, connecting tube between uterus and cervical vagina, the valve guarding the connection between the vaginal parts and absence of coxal organs are similar to ixodids [9]. It was shown that the mode of feeding in nymphal and adult females are rapid similar to argasids, but that blood meal concentration occurs via the Malpighian tubules [6].

The morphological data thus far generated for *N. namaqua* were mostly derived from adult females [4]–[7]. The male for *N. namaqua* had not been found yet and led to speculation on whether females were parthenogenic, males were secretive or whether the sex ratio was disproportionate [10]–[11]. To date, the morphology of the larvae and nymphae had not been investigated yet and the question arose as to which characteristics will be shared with hard and soft ticks. The problem of interfamily relationships within the tick families would not be resolved until information on the structure and biology of the immature stages and the male tick had become available [7]. While molecular systematic studies have addressed this problem, questions regarding the biological and morphological affinity of the different life stages of *N. namaqua* remain. We investigated these issues by description of the larvae, nymphae, female and male ticks from a morphological perspective.

## Results

### Description of Larva (Fig. 1 A–M)

#### Biology

Two field-collected engorged females as well as two females mated in the laboratory each laid small batches of 80–150 eggs. Eggs hatched within 14 days and yielded viable larvae that were similar in morphology to previously collected dead larvae [6]. One female tick fed successfully to repletion between two ovipositions.

**Figure 1:**
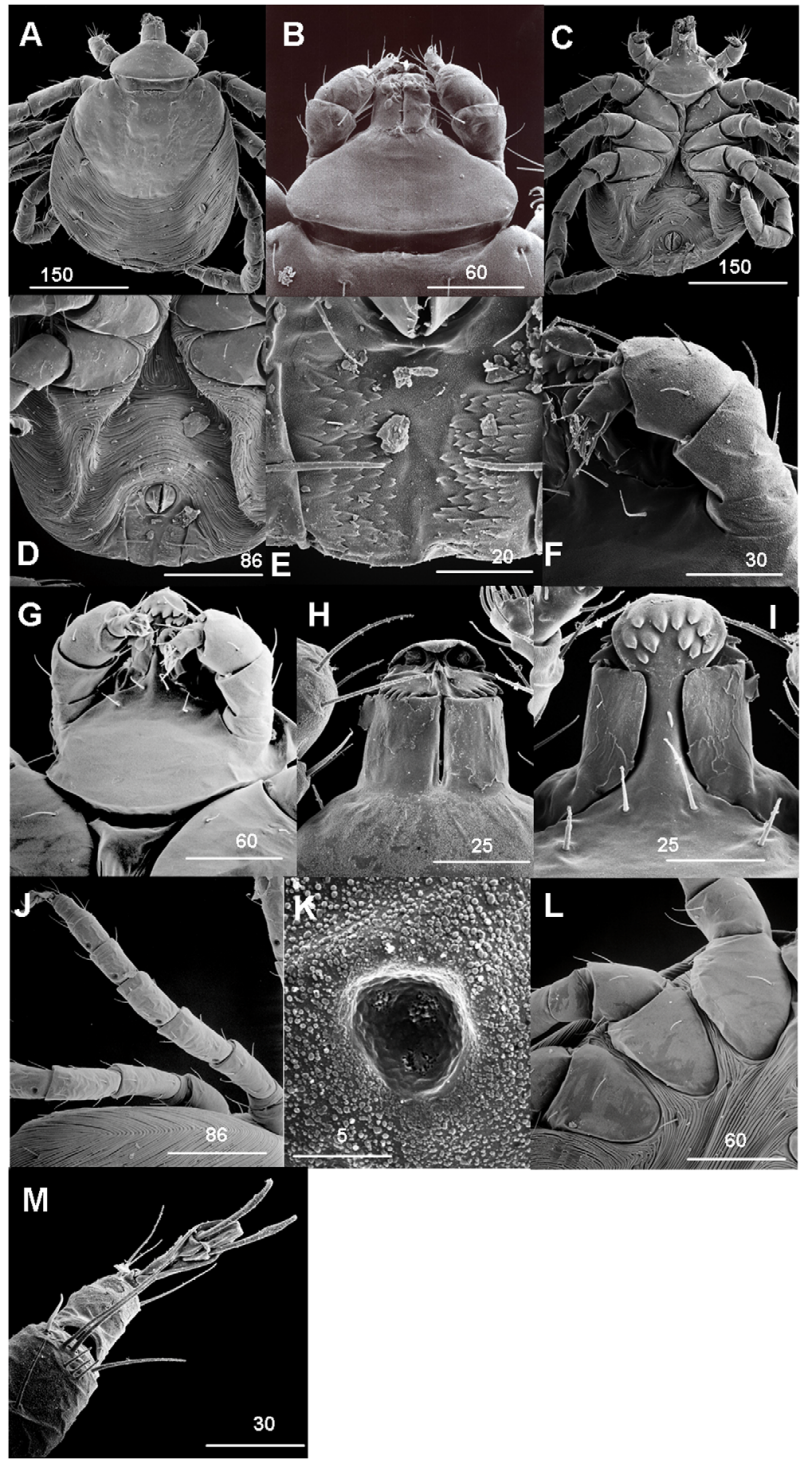
*N. namaqua* larva. A) Scanning electron micrograph of dorsal integument. B) Dorsal basis capituli. C) Scanning electron micrograph of ventral integument. D) Posterior venter. E) Anal plate. F) Palps. G) Ventral basis capituli. H) Hypostome dorsal. I) Hypostome ventral. J) Pores in origin of femur, metatarsus, tibia and tarsus. K) Leg pore structure. L) Coxae. M) Haller’s organ and claws. Scale bars are indicated in µm.

#### Body

Sub-circular, 0.46 mm from posterior margin to apex of hypostome.

#### Dorsum

(Fig. 1A)**:** **Scutum:** length 0.22 mm, breadth 0.25 mm, anterior 1/3 demarcates the lateral body margin; margins converge making rounded posterior margin. **Scutum**; sclerotized, with slightly roughened surface for 2/3 of posterior region, total of 8 setae; 4 present on the anterior margin (Fig. 1B) (some setae broken on Fig. 1A). Cervical grooves and eyes absent. **Alloscutum:** covered by longitudinal fine dense but separated striations, running parallel from one lateral margin of body to the other; 4 pairs of setae on postero-lateral margin and 3 pairs of medial setae (Fig. 1A); some setae broken.

#### Venter

(Fig. 1C, 1D, 1E): Integument as on the dorsum; striations curve anteriorly around the anal pore, diverging towards posterior body enclosing anal plate; anal opening with pre-anal ring (Fig. 1D); 4 pairs of main setae; one pair located anterior to pre-anal ring, one pair on anal valves, one pair on postero-lateral margins of pre-anal ring, the 4^th^ pair long and located anterior to the posterior body margin with seta directed towards the other across the anal plate. Unique presence of anal plate consisting of rows of denticles or hooklets on two sides, separated by the median post-anal groove extending to the posterior margin (Fig. 1E). Anal plate visible from the dorsal side situated at the most posterior body margin body (Fig. 1A).

#### Capitulum

(Fig. 1A, 1B, 1F, 1G, 1H, 1I): **Palps** (Fig. 1F).: short (0.07 mm), 4 segments, segment 1 short, 2 longest, 3 large and arising from segment 2; segment 4 inserted in terminal aperture of segment 3. Setae absent in segment 1, segment 2 and 3 each with 2 pairs of dorsal and ventral setae, segment 4 with a total of 10 setae, a tuft of 3 setal pairs terminally situated. **Basis capituli**: lateral margins triangular in dorsal view with broader angles and the posterior margin slightly convex with no lateral projections (Fig. 1A and 1B). Ventrally, posterior margin more convex with antero-lateral projections; two pairs of posthypostomal setae present (Fig. 1G); auriculae and cornua absent. **Hypostome** (Fig. 1H): Dorsally, cheliceral gutters with cheliceral denticles long and prominent. A pair of large “pits” protected by a membranous hood present at the apex of the hypostome. Ventrally, hypostome ball-like structure at its apex, with 11 prominent denticles arranged in 2 rows (Fig. 1I). Rudimentary or minute denticles absent. Five posthypostomal setae present; two long pairs and a single short one.

#### Legs

(Fig. 1J, 1K, 1L): Long and slender, carrying a pore at the origin of the femur, metatarsus, tibia and tarsus, each pore located at proximal portion of these segments (Fig. 1J). Pores located only on the dorsal side of legs. At higher magnification the pore shows 3 receptor-like structures in the interior (Fig. 1K). **Coxae:** Wide U-shape at the base, closely located to each other, no coxal spurs, 2 setae present on each coxa and 2–6 present on other segments of legs (Fig. 1L).

#### Haller’s organ

(Fig. 1M): 8 setae, 4 short and 4 long located on posterior section; setae not present on anterior section, 2 sensilae seen inside the organ pit. A pair of claws and pulvilli present on the ambulacrum.

### Description of the Nymph (Fig. 2 A–J)

#### Body

**Figure 2:**
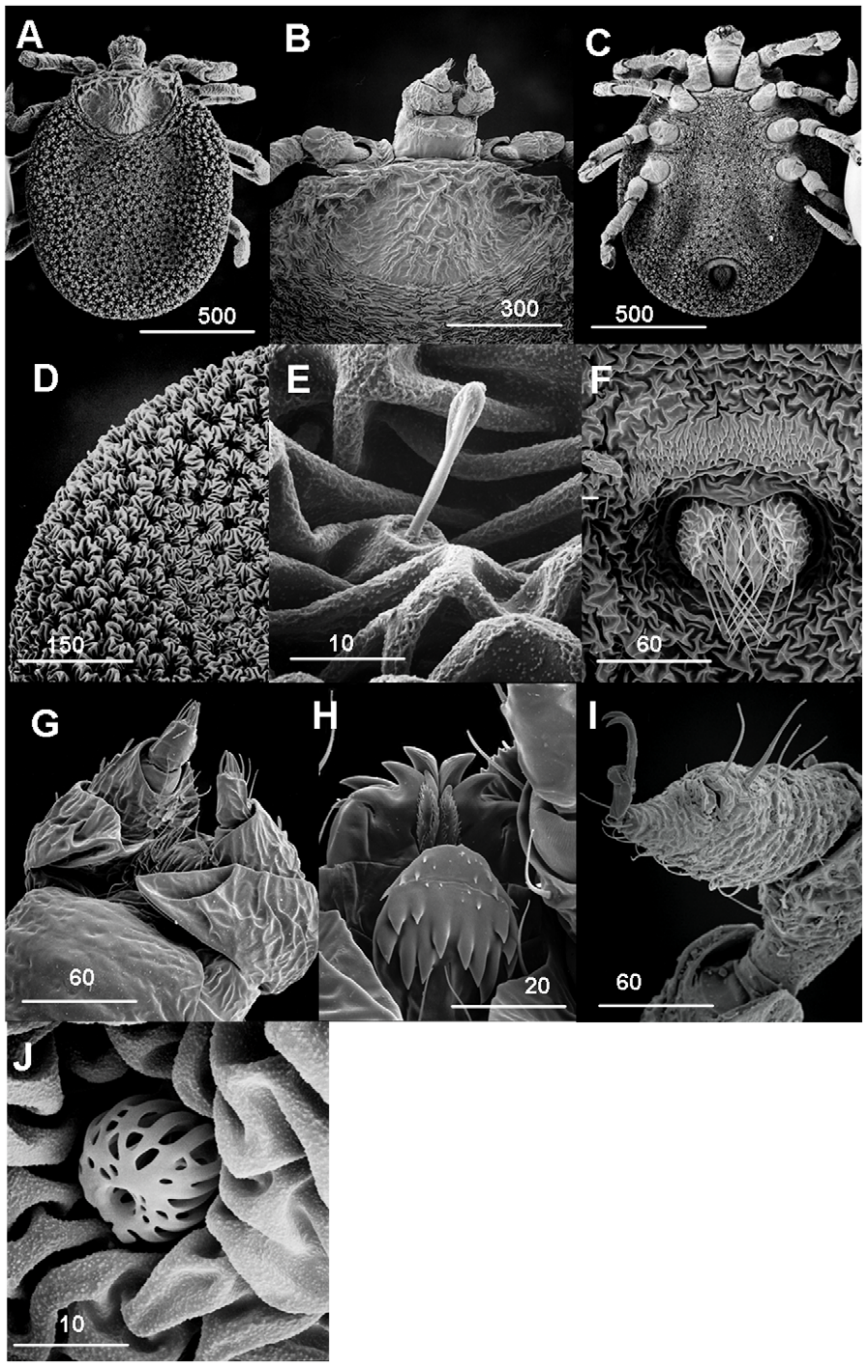
*N. namaqua* nymph. A) Scanning electron micrograph of dorsal integument. B) Scanning electron micrograph of dorsal basis capitulum. C) Scanning electron micrograph of ventral body integument. D) Integument. E) Setae in rosette pits. F) Anal pore. G) Palps. H) Hypostome ventral. I) Haller’s organ and claws. J) Spiracle plate. Scale bars are indicated in µm.

Idiosoma circular, 1.58 mm long from posterior margin to apex of hypostome (Fig. 2A). Gnathosoma arising anterior of body, bordered laterally by coxae 1. Eyes absent. **Dorsum** (Fig. 2A and 2B): **Pseudoscutum** broad, posterior margin circular, integument surface with elevation forming a network of irregular shallow larger compartments anteriorly extending to the centre, length 0.55 mm, breadth 0.35 mm. **Alloscutum** (Fig. 2A, 2C, 2D and 2E): dorsal and ventral surface highly convoluted with dense elevated rosettes, marginal rows of setae and some scattered on the rest of the integument within pits of rosettes.

#### Venter

(Fig. 2C and 2F): Pre-anal groove a demarcated half-moon shaped structure bordering the anterior of anal pore, posterior margin of groove with about 5 rows of closely spaced dentate integumental projections. Anal pore has a tuft of long fine setae, about 9 on each valve crossing each other and extending to the outside; pre-anal ring as in Figure 2F.

#### Capitulum

(Fig. 2B, 2C, 2G, 2H): **Basis capituli** (Fig. 2B): rectangular in shape with width twice the length; coxae 1 arising at its base. The integument at the anterior half convoluted, posterior smooth; setae absent. Ventral view (Fig. 2C): trapezoid in shape with its two lateral sides bordered by coxae 1. **Palps** (Fig. 2G)**:** Four segments as described for female below. The enfolding “crib” in segment 1 prominent. Setae absent on segments 1 and 3; 2 pairs on segment 2 while segment 4 bears a tuft of 7 setae. **Hypostome** (Fig. 2H): cheliceral digits outer and inner articles with prominent curved teeth; a pair of stylets with small denticles located anterior to cheliceral digits. Hypostome bluntly rounded apically, denticles arranged in two rows from top to bottom, each row with 3 large ones; small and rudimentary denticles located towards apical region. Two long posthypostomal setae present.

#### Legs

(Fig. 2B, 2C): Long, slender, beaded, orange colour in live specimen. Coxa 1 borders capitulum anteriorly and laterally; part of coxa 2 contiguous to 1; 2 and 3, and 3 and 4 separated from each other. Coxa 1 with large outer spur, coxae 2, 3, 4 unarmed. Setae absent on coxae. Leg segments articulate by ball and socket joints.

#### Haller’s organ

(Fig. 2I): 3 pairs of long and one pair of short (broken) setae on the posterior and anterior sections, respectively. Pulvilli present on ambulacrum, but reduced.

#### Spiracle plate

(Fig. 2J)**:** Positioned immediately posterior to coxa 4 surrounded by convoluted integument, arising as a rounded perforated “fenestrated” plate surface with a circular opening at the apical surface.

### Re-description of the Female (Fig. 3 A–E)

#### Basis capituli

**Figure 3:**
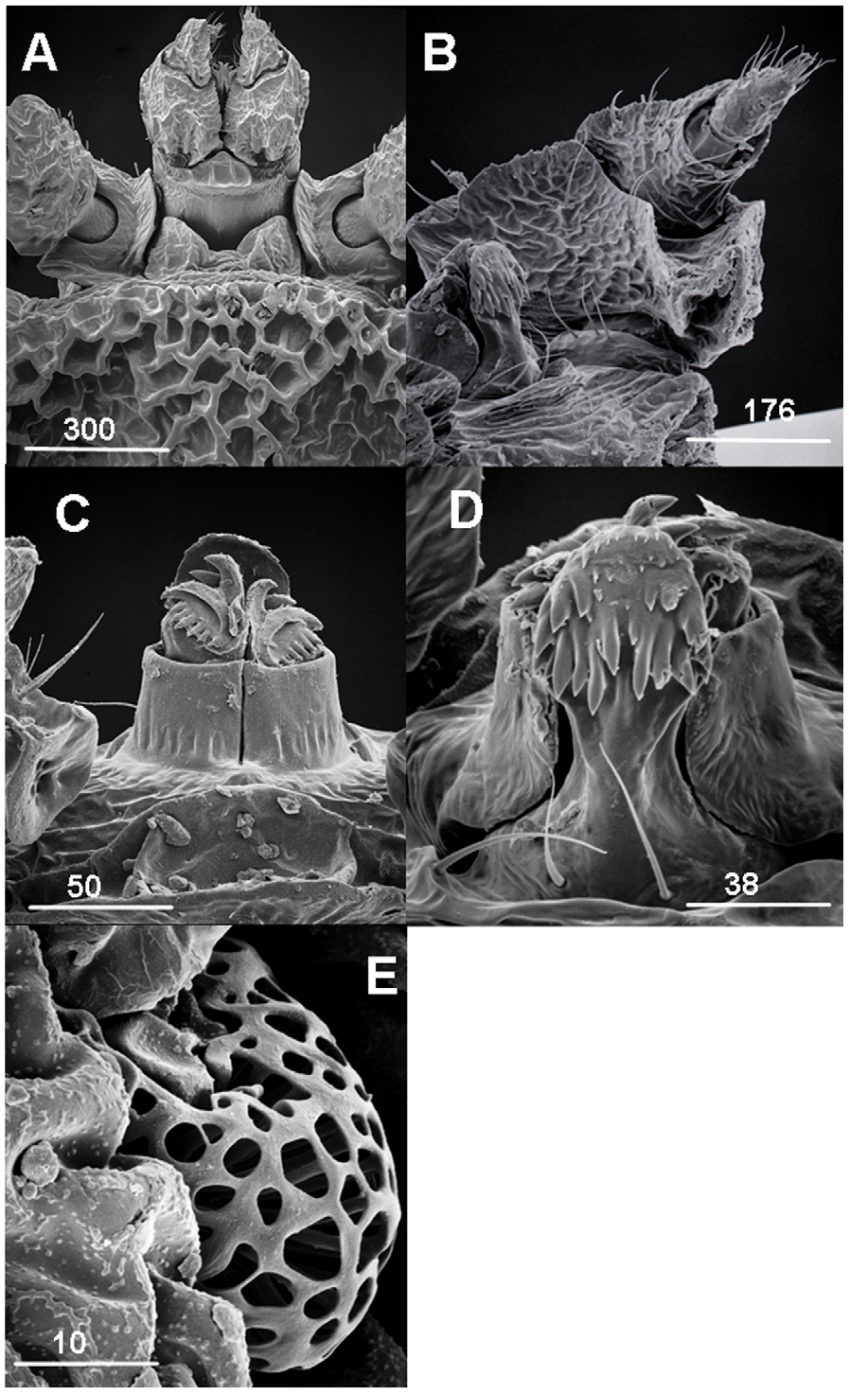
*N. namaqua* female. A) Basis capitulum. B) Palps. C) Hypostome dorsal. D) Hypostome ventral. F) Spiracle plate. Scale bars are indicated in µm.

(Fig. 3A)**:** Features as described [7], but setae absent.

#### Palps

(Fig. 3B)**:** 4 segments, segment 1 previously described [7] as a massive structure, internal surface emarginated to form a crib for the posteriorly directed segment 4 (and not segment 3 as previously described [7]). Segment 2 less than half size of segment 1 arises from the anterior margin of segment 1. Segment 3 arises from within segment 2, the smallest and bears the elongated segment 4; 5 setae on segments 1 and 2; no setae on segment 3, segment 4 bears a tuft of setae about 15 in number.

#### Hypostome

Dorsal view (Fig. 3C): chelicerae with outer and inner digits; ventral view (Fig. 3D): hypostome bluntly rounded apically, large distinct denticels, totalling about 13, arranged in two rows from top to bottom, bottom row of denticles the largest. Dental formula remains indeterminate [7]. Small rudimentary rows of denticles present near the apex. A pair of long posthypostomal setae present.

#### Spiracle plate

**Figure 4:**
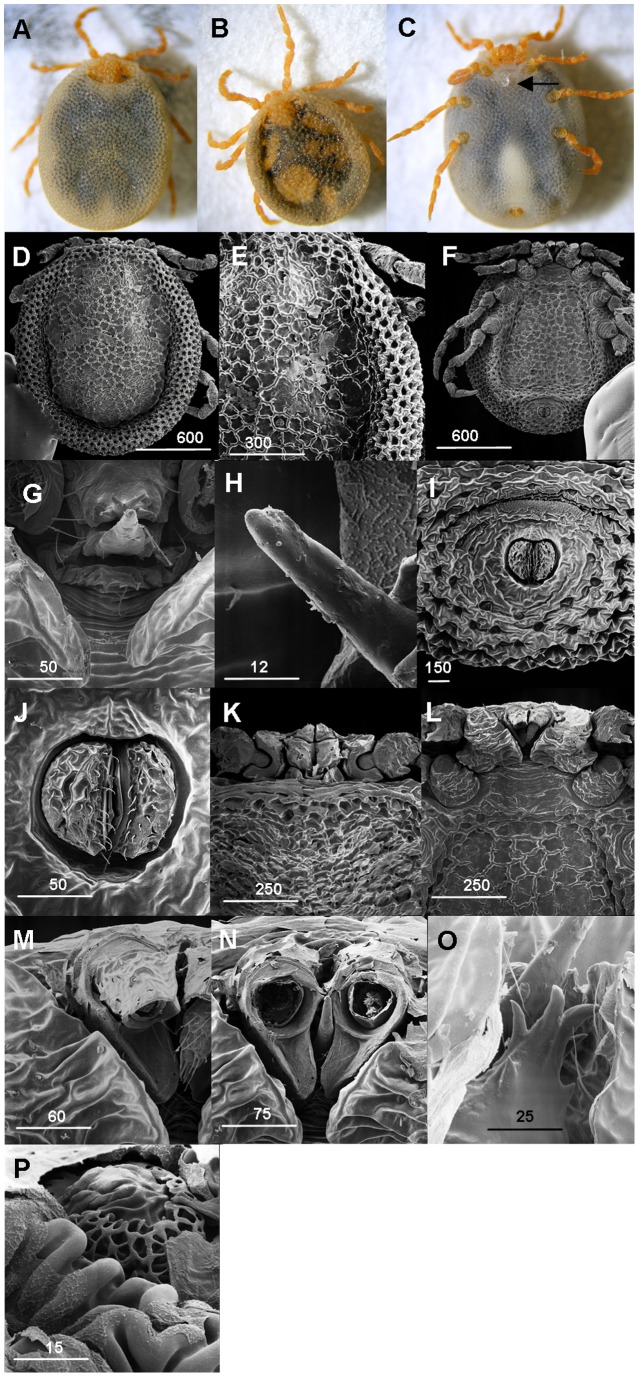
*N. namaqua* male. A) Stereo micrograph dorsal view *N. namaqua* female. B) Stereo micrograph dorsal view *N. namaqua* male. C) Stereo micrograph ventral view *N. namaqua* female; spermatophore deposited in genital pore (arrow). D) Dorsal view male. E) Integument dorsum male. F) Ventral view male. G) Genital area male. H) Genital area male. I) Preanal groove male. J) Anal pore male. K) Capitulum dorsal view male, including coxae 1, proximal segments of leg 1, pseudoscutum integument. L) Capitulum ventral view male, including coxae1 and 2. M) Capitulum dorsal view male, one palpal segment 4 removed. N) Capitulm dorsal view male, both palpal segments 4 removed. O) Hypostome male, chelicerae. P) Spiracle plate. Scale bars are indicated in µm.

(Fig. E): Fenestrated plate as previously described [8]; structure differ from nymph (Fig. 2J) and male (Fig. 4R).

### Discovery of *N. Namaqua* Male

Of the engorged adult ticks collected a pair was found in copula i.e. positioned venter to venter. They were transferred to the laboratory at ARC-OVI and immediately kept under controlled conditions (23°C, 75% RH). The pair disengaged but re-attained the mating position. The engorged female later laid eggs and the egg batch hatched into viable larvae. A total of 6 flat and partially fed adults were applied to feed on a lizard and fed to engorgement as reported previously [6]. Again, one pair, an engorged female and a morphologically similar tick of smaller size, were found in a mating position. The ticks were separated and examined using stereomicroscopy (Fig. 4A and 4B). After mating, a completely developed spermatophore (bilobed) was found at the opening of the genital pore of the engorged female (Fig. 4C). The female was allowed to oviposit and the eggs hatched into viable larvae. We describe below the smaller tick which was confirmed to be a male of *N. namaqua*.

### Description of Male (Figs. 4 B, 4D–4Q)

Length from posterior margin to apex of palps 1.66 mm; width 1.44 mm at level of leg 4. Body outline oval; capitulum arising anterior of body, bordered laterally by coxae 1. Colour of live specimen: legs orange, body yellowish/orange at margins and ornate pseudoscutum, black with large orange spots (Fig. 4B).

#### Dorsum

(Fig. 4D, 4E) **Pseudoscutum:** 1.44 mm long, 1.03 mm wide at mid length, almost rectangular shape, covers 62% of central dorsal surface, its anterior margin demarcates the anterior margin of the body, the margins run posteriorly parallel to the lateral margins of the body, then converge making a semi-circular posterior margin. The integument surface with elevation forming a network of irregular shallow larger compartments. Eyes absent. **Alloscutum** (Fig. 4D, 4E): Integument highly convoluted, forming closely spaced pits surrounded by elevated rosettes.

#### Venter

(Fig. 4F): Integument structure similar to dorsal; genital pore (Fig. 4G) situated between coxae 1, a transverse slit, length 0.085 mm, width 0.036 mm, external margins of lips dentate. Ventral plate (Fig. 4F) extends from posterior to coxae 2 diverging posteriorly then converging medially at level anterior to pre-anal groove. Pre-anal groove (Fig. 4F, 4I) slightly anterior of anus, a demarcated curved-shaped structure with numerous closely spaced dentate projections. The anal pore near posterior body margin; pre-anal outline as illustrated, each valve with about 17 fine short setae (Fig. 4J).

#### Capitulum

(Fig. 4K–4O)**: Basis capituli** dorsally (Fig. 4): **Cornua**: long subtriangular anteriorly directed on each side. Ventral view obscured by coxae 1 (Fig. 4L). **Palps**: 0.17 mm long, width 1.66 mm; palpal segments 2 with large ventral sheath, a spade-like structure extending to a level just anterior to palpal segments 4 and forming a crib for it (Fig. 4M, 4N); segments 4 bear 12 setae, 7 at its apices (Fig. 4M). Hypostome: chelicerae with prominent denticles (Figure 4O). An outgrowth on the chelicerae broad at its base, forming a unique rod-like structure directed upwards similar to spematodactyl, 0.064 mm long (Fig. 4G, 4H).

#### Legs

(Figs. 4F, 4K): As described for the female [7]; long, slender, beaded, coxae 1 arising from anterior body margin and closely adjacent to capitulum; part of coxa 2 contiguous to coxa 1; 2 and 3 and 3 and 4 separated. Leg segments articulated by ball and socket joints (Fig. 4K).

#### Spiracle plate

(Fig. 4P): located immediately posterolaterally to coxa 4, fenestrated plate except upper surface, differt from the nymph (Fig. 2J) and female (Fig. 3E) spiracles.

## Discussion

The current study described the morphology of *N. namaqua* larvae, nymphae and males for the first time. In addition, key aspects of the female tick not described in detail or which gave discrepancies in previous studies were re-described [4], [7]. This included the hypostome and chelicerae and the difference in the number of palpal segments [4], [7]. The male was also shown to exist and to copulate with females, leading to successful egg laying and viable progeny.

Larvae possess a sclerotized scutum similar in size and appearance to that observed in larval ixodids. Given the basal position of *N. namaqua* to the other tick families [6], we propose that a sclerotized scutum as observed in all life stages of the Ixodidae was present in the last common ancestor to all ticks and was derived from a more ancestral parasitiform character [12]. This feature was retained in *N. namaqua* larvae and ixodid larvae, nymphae and adults, but was lost in argasid nymphae and adults and only partially retained in argasid larvae as dorsal plates and *N. namaqua* adults and nymphae as a pseudoscutum. Features that are unique to larvae and which were possibly lost in the main tick families and *N. namaqua* nymphae and adults include the dentate anal plate and the pores on the dorsal side of the legs.

Unlike argasids, all life stages can climb the smooth walls of the plastic tubes in which ticks were kept. The presence of pulvilli on the ambulacrum corroborates this and is another feature shared with ixodids [13]. This is a feature most probably associated with their habitat lifestyle of frequenting rocky ledges, cliffs and crevices [6], [7]. The fourth segment of the palps is terminal as seen for the adults and is similar to argasids [4]. The pores located only on the dorsal side of the larval legs that possess 3 porous receptor-like structures are probably nonsetal sensillar cupules normally found on the chelicerae, pedipalps and legs of the Acari [13]. These could be strain, sound, substrate vibration, gravity or chemoreceptors. Being on the dorsal side only may also indicate involvement in photoreception. The anal plate is a feature unique to *N. namaqua* larvae and the denticles or hooklets suggests that this structure might function as anchor for the larvae, probably during host attachment and when climbing smooth surfaces. Similarly, the nymphae, males and females possess dentate integumental projections on the anterior side of the anal pore that could function in a similar manner. The larval, nymphal and female hypostome possess a ball-like bluntly-rounded apical structure that is unique to *N. namaqua*.

Like the nymphae and adults, larvae show characteristics shared with ixodid and argasid ticks as well as features unique to this lineage. Overall, the larvae show a greater similarity to ixodid ticks and little resemblance to nymphal or adult *N. namaqua* ticks due to the sclerotized scutum and absence of defined ball and socket joints. The larvae collected from different species of murid rodents and assigned to *Nuttalliella* sp. (*N. namaqua*) on the basis that they resemble the adult tick features [14], do not conform to the morphological characters of the species as described above. The latter study collected larvae from murid rodents, while a gut meal analysis from a field collected female tick indicated that it had fed on lizards [6], [14]. It would therefore seem that *N. namaqua* is a multi-host tick, a character trait shared with argasids [3].

*N. namaqua* larvae differ significantly from its other life stages; the nymphae have essentially the same morphology as females, a feature that is found in many argasid species [3], [10]–[11]. The current study helped to resolve some discrepancies in the female morphology raised in previous studies. Hard and soft ticks have four palpal segments, while the presence of only three segments in *N. namaqua* was considered to be a unique feature of this lineage [7]. However, the original description indicated four segments [4]. The current study also found four segments, the first which is reduced in the larvae similar to many ixodids. We, therefore, contend that four palpal segments are an ancestral trait in the Ixodida. The first segment in the nymphs and the adults is a massive structure of which the internal surface is emarginated to form a crib (unique in *N. namaqua*) into which the posteriorly directed segment four can be placed possibly for protection (behavioral observation of living specimens). Both nymphae and adults possess anal valves with numerous setae which is absent in the larvae as well as in hard and soft ticks. Feeding females secrete water anally via the Malpihian tubules [6], and the setae may play a role in this. It also suggests that larvae might secrete water in a different manner.

The current study also resolves the question regarding the existence of the male tick. Mating occurred off the host as observed in argasids and Prostriate ticks [10]–[11]. The bilobed spermatophore that was observed after copulation is similar to that described for argasids [13]. Oviposition was similar to argasids, in that small egg batches of only a few hundred eggs were laid and females are able to lay more than one egg batch, with feeding in between [15]. Characteristic male features include: presence of pseudoscutum over most of dorsum, an outgrowth on the chelicerae broad at its base, forming a unique rod-like structure similar to the spermatodactyl found in mites [16], medial extension of palpal segment 2 forming a large ventral sheath that acts as a crib for segment 4. The spermatodactyl occur in the mesostigmatans, notably, the Dermanyssina and Heterozerconina as an outgrowth of the chelicera and transfer sperm during copulation [16]. In the case of the *N. namaqua* male, it is likely that the hypostome were modified to perform this function.

### Conclusions

All life stages show some morphological characters similar to hard and soft ticks, as well as characters unique to *N. namaqua*. The description as the “missing link” between the hard and soft tick families [4] remains a viable concept which is supported by molecular systematics and the unique larval characters support the notion that this species is a “living fossil” [6]. *N. namaqua* will therefore remain a good model for the study of ancestral characters related to the evolution of blood-feeding in ticks.

## Materials and Methods

### Ethics Statement

Experiments related to the feeding and maintenance of tick colonies involving the use of animals were approved by the ARC-OVI Animal Ethics Committee; Approval Number AEC12-11. All necessary collection and transport permits were obtained from the Veterinary Authorities (Permit number: SP2011/02/02/01).

### Tick Collection and Colonies

*N. namaqua* adults, nymphae and larvae were previously collected by us [6]. A second tick collection was made during August-September 2011 from the same locality. Engorged females were allowed to lay eggs and viable larvae were subsequently obtained. Preserved tick specimens (adult, nymph, larvae) in 70% ethanol were micrographed using a Zeiss V-20 stereomicroscope and also processed for scanning electron microscopy. Three larvae were processed, one from the ground collection which was confirmed to be *N. namaqua* [2], and two from the larval batches produced from engorged females.

### Scanning Electron Microscopy

Due to the fragile nature of this particular species of soft-bodied tick, conventional methods for cleaning could not be used [17]. Provisional test samples of an adult and nymph simply broke up as the glue was removed from the sample. Ticks were subsequently placed in porous specimen capsules (Agar Scientific, Essex, England) and processed through all solutions. Specimens were re-hydrated from 70% ethanol to ddH_2_O and processed through ultra-sonication (5×2–3 second bursts) in a Bransonic 2210 ultrasonic cleaning bath (Branson Ultrasonics Corporation, Connecticut, USA). Re-dehydration was through an ascending series of ethanol (30, 50, 70, 90, 95 and 3×100%) for 1 hour per step. Specimens were dried in an E3100 Jumbo Series II critical point drying apparatus (Polaron Equipment Limited, Watford, England) from 100% ethanol through ℓ-CO_2_ and mounted individually onto conical brass viewing stubs using Japan Gold Size (Winsor & Newton, London, England) as an adhesive. Mounted specimens were sputter-coated with gold in a Balzers-020 sputter coating apparatus (Balzers Union Ltd., Liechtenstein) and viewed in a Hitachi S-2500 scanning electron microscope (Hitachi Ltd., Tokyo, Japan) at 8 kV accelerating voltage. As the specimens had been fixed in 70% ethanol, some detritus had adhered permanently to the specimen and could not be removed using this method.
